# Association of Virtual Nurses’ Workflow and Cognitive Fatigue During Inpatient Encounters: Cross-Sectional Study

**DOI:** 10.2196/67111

**Published:** 2025-04-22

**Authors:** Saif Khairat, Jennifer Morelli, Wan-Ting Liao, Julia Aucoin, Barbara S Edson, Cheryl B Jones

**Affiliations:** 1 Carolina Health Informatics Program, University of North Carolina at Chapel Hill, Chapel Hill, NC, United States; 2 School of Nursing, University of North Carolina at Chapel Hill, 438 Carrington Dr, CB 7460, Chapel Hill, NC, 27519, United States, 1 919-843-6761; 3 Cecil G. Sheps Center for Health Services Research, University of North Carolina at Chapel Hill, Chapel Hill, NC, United States; 4 Lineberger Cancer Comprehensive Cancer Center, University of North Carolina at Chapel Hill, Chapel Hill, NC, United States; 5 Virtual Care Center, UNC Health, Chapel Hill, NC, United States

**Keywords:** virtual nursing, telemedicine, cognitive fatigue, eye-tracking technology, eye tracking, eye, nursing, virtual, cross-sectional study, workflow, inpatient, fatigue, pupil size, pupil, tracking, USA, United States, design, cognitive, virtual care, nurse, delivery model, technology, communication, EHR, electronic health record, virtual nurse

## Abstract

**Background:**

The virtual nursing delivery model enables the provision of expert nursing care from a remote location, using technology such as audio and video communication, remote monitoring devices, and access to electronic health records. Virtual nurses spend an extensive amount of time on computers to provide care, and little is known about how this workflow may affect and contribute to cognitive fatigue.

**Objective:**

This study aimed to use eye tracking technology and pupil size variation to determine instances of virtual nurse cognitive fatigue during their typical workflow.

**Methods:**

This study examined the virtual nursing workflow by recording and analyzing virtual nurse encounters using eye tracking. This cross-sectional study was conducted during regular 12-hour shifts at a major Southeastern health center in the United States.

**Results:**

The study found that 75% (22/29) of virtual nursing encounters demonstrated a first fatigue instance at 9.8 minutes during patient discharges and at 11.9 minutes during patient admissions.

**Conclusions:**

This study provides valuable insights into virtual nursing workflow design and how it may impact the cognitive fatigue levels of nurses providing inpatient virtual care.

## Introduction

Over 25% of US registered nurses may quit by 2027 due to burnout and challenging work environments, contributing to high nurse turnover rates [1]. Nurses experiencing burnout are five times more likely to delay care (eg, documentation and patient discharge) than nurses without burnout, leading to long patient wait times [2]. Incomplete nursing documentation threatens patient care and safety [3]. Patient home medication review, an essential element of patient admission, is commonly missed due to fatigue, a lack of training, competing tasks, and insufficient time [4,5]. A lack of medication review can lead to medication errors, readmissions, and increased healthcare costs [6-8]. In addition, nurse burnout can result in delays in care and promote inpatient complications and poor patient satisfaction [9-11].

Healthcare systems in the United States are swiftly adopting a novel care delivery model, virtual nursing [12]. Virtual nursing provides bedside nurses with remote nursing expertise to reduce their workload and alleviate work-related fatigue [13]. Virtual nursing aims to mitigate burnout by reducing the workload and improving documentation completeness at admission and patient education at discharge [14].

A key consideration in implementing this emerging care delivery model is understanding workflow design, which protects virtual nurses from the unintended consequences of continuous virtual patient encounters [15]. Extended screen time and the use of electronic health records (EHRs) were associated with an increase in burnout and fatigue in bedside nurses [16]; however, the association between continuous screen time and virtual nurses’ fatigue levels, a key factor to the sustainability of this model, is unknown. Pupillometry data, which measures the change in pupil size, measures cognitive fatigue [17]. This study examined the association between using virtual nursing and virtual nurse fatigue using eye-tracking technology.

Eye-tracking technology provides real-time insights into cognitive workload and cognitive fatigue by recording eye movements during different states and activities. Pupil size in particular can be an indicator of cognitive workload, where an increase in pupil dilation corresponds to information processing [18,19]. In instances of cognitive fatigue, pupils exhibit less dilation over time or decreased response to stimuli. As the mental effort to process information increases, pupils constrict, indicating mental fatigue or exhaustion [20].

## Methods

### Participants, Device, and Measurements

We conducted a cross-sectional study of virtual nurses during regular 12-hour shifts at a Southeastern tertiary hospital. We observed virtual nurses during four times of interest (TOIs): preparation (eg, chart review), admission encounter, discharge encounter, and miscellaneous (eg, documentation). All virtual nurses were previously bedside nurses at the same hospital. At the time of observation, six nurses were on staff at the Virtual Care Center during day shifts. Four nurses were observed over a period of 5 days.

We used a noninvasive screen-mounted eye-tracking device (Tobii® Pro Spark, Tobii AB®, Stockholm, Sweden) to capture pupillometry data of the virtual nurses during their shifts. Our target number of observations (ie, sessions) was based on evidence that suggests a sample size between 10 and 50 observations suffices for reliable and valid results [21-23].

The eye-tracking device was set up at the virtual nurses’ workstations on the monitor that displays activity in the EHR. Prior to recording sessions, virtual nurses were oriented with the eye-tracking device and instructed to complete their tasks as they normally would. An initial calibration session was completed with each virtual nurse before recordings began to ensure data quality. Due to fluctuations in shifts and general availability, two of the four nurses had a higher number of recordings and TOIs captured. To help mitigate this as a confounder, we focused our analysis on the encounters and TOIs rather than the individual nurse level.

### Ethical Considerations

The Institutional Review Board (IRB) at the University of North Carolina approved this study under IRB number 23‐0859. Information related to the study was provided to nurses prior to participation, and they were informed they could decline to participate or withdraw at any time. All participating nurses provided verbal consent to the study team members prior to eye-tracking recording. Participating nurses were given a US $25 gift card to thank them for their participation. All data collected was anonymized following data collection to protect participant confidentiality.

### Study Outcomes

The primary study outcome was cognitive fatigue measured as changes in pupil size. Secondary outcomes were subgroup analysis of fatigue by nursing tasks and patient acuity.

### Statistical Analysis

Eye-tracking data were exported, and fatigue counts per minute were calculated by flagging instances where the average pupil size was below the calculated threshold. The fatigue threshold for each recording was calculated by subtracting 1.5 times the SD from the mean pupil size. The Shapiro-Wilk test was used to test for normality and the Mann-Whitney or Kruskal-Wallis tests were used to compare continuous variables. We conducted all analyses with statistical software (RStudio version 2023.12.1; Posit Software, PBC).

**Figure 1.**
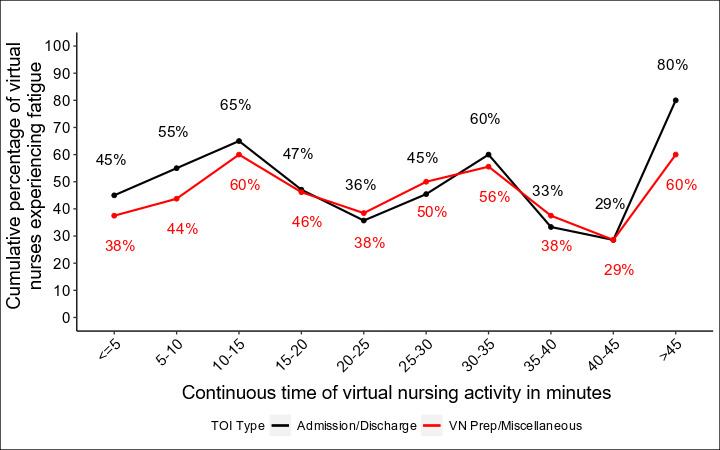
Proportion of virtual nurses experiencing cognitive fatigue during virtual nursing sessions over time by 5-minute intervals. TOI: time of interest.

**Figure 2.**
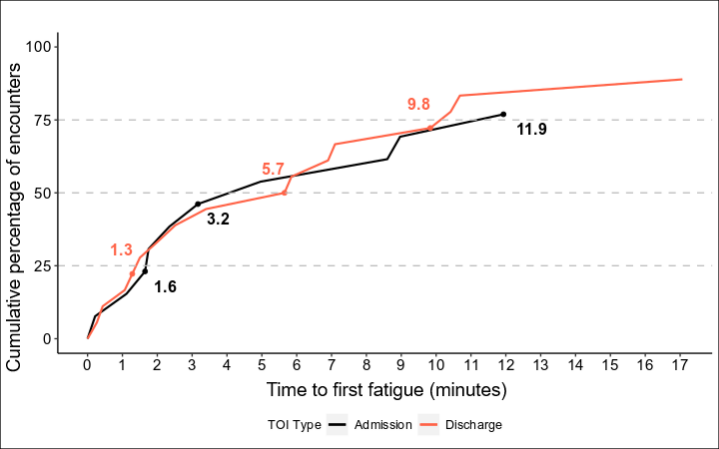
Cumulative percent of virtual nurses experiencing their first cognitive fatigue instance during admission and discharge times of interest (TOIs).

**Figure 3.**
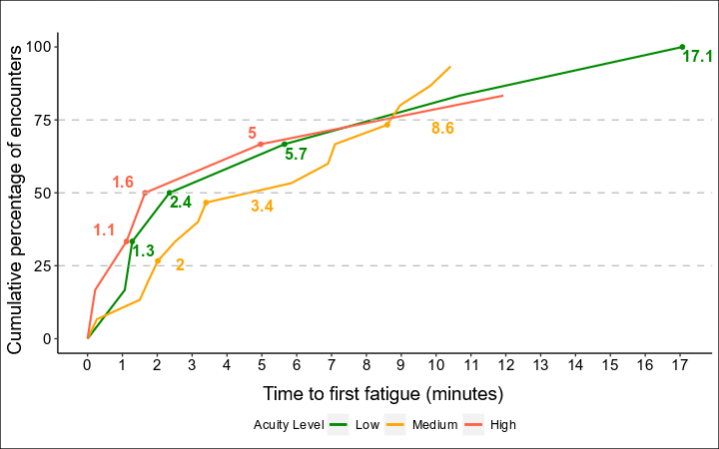
Cumulative percent of virtual nurses experiencing their first cognitive fatigue instance for all times of interest (TOIs) by patient acuity.

To generate figures for visualization, we examined the instances of fatigue in a given recording and tracked them over time. We were most interested in comparing the instances of fatigue during patient encounters (admissions and discharges) and other virtual nursing tasks (preparation and miscellaneous). In Figure 1, the x-axis is represented in continuous time groups of 5 minutes, while the y-axis represents the percentage of virtual nurses who experienced an instance of fatigue in a TOI. As encounter times differ and often decrease over time, the number of recordings that meet a given duration also decreases, leading to a smaller ratio. However, this does not fully explain the higher percentage, as it can also be attributed to a greater number of virtual nurses experiencing fatigue as time progresses. In Figure 2, we note the time to first fatigue in minutes, specifically in patient admission and discharge TOIs, and note the cumulative percentage of virtual nurses who have experienced an instance of fatigue by that period of time. In Figure 3, we compare all recordings of patient encounters and look instead at first fatigue by the level of patient acuity.

To determine patient acuity, we used a combination of factors, including patient age, expected or actual length of stay, principal hospital problem, and severity of the patient unit. These factors were weighted for each patient admission or discharge encounter to determine an acuity rating of low, medium, or high. We used data from the Center for Disease Control National Hospital Discharge Survey [24] to inform this process. The age groups of 18‐44 years, 45‐64 years, and >65 years were utilized, with the greatest weight given to patients over 65 years of age. The expected or actual length of stay was categorized into groups of 1‐3 days, 4‐6 days, and >7 days, with individuals staying or expected to stay greater than 7 days at the highest acuity. In cases where the principal hospital problem was given (27/29 instances), this was compared to the Center for Disease Control data; the principal hospital problem was compared to the Center for Disease Control data along with the patient’s age and length of stay to determine if the patient had lower or higher than the average length of stay. Finally, the severity of the patient unit was taken into consideration, with short-stay units given the lowest acuity, general medical-surgical units given medium acuity, and intensive care units or critical care units given the highest acuity scores.

## Results

A total of 22 recordings and over 14 hours of recording time were analyzed, encompassing 83 distinct virtual nursing encounters, Table 1. Of the 83 TOIs, 11 (13%) were admission encounters, 18 (22%) were discharges, 36 (43%) were miscellaneous, and 18 (22%) were preparation tasks. The average (SD) duration was 17 minutes 48 seconds (5.3 minutes) for admissions and 17 minutes 53 seconds (8.9 minutes) for discharges. All four virtual nurses were female, with an average (SD) of 26.3 (7.9) years of nursing experience.

**Table 1.** Distribution of workflow tasks for study participants.

	Total observed time	No. of recordings	No. of admissions	No. of discharges	No. of miscellaneous sessions	No. of preparation sessions
Nurse A	6 hours, 8 minutes	11	6	5	18	7
Nurse B	1 hour, 4 minutes	1	0	2	2	1
Nurse C	3 hours, 2 minutes	1	1	3	8	7
Nurse D	4 hours, 27 minutes	9	4	8	8	3
Total	14 hours, 41 minutes	22	11	18	36	18

The average (SD) number of cognitive fatigue instances varied by TOI such that admission encounters had 16.9 (16.4) fatigue instances, discharges had 15 (15.9) fatigue instances, preparation had 4.7 (8.3) fatigue instances, and miscellaneous had 0.42 (1.3) fatigue instances.

Spikes in cognitive fatigue occurred approximately every 15 minutes (Figure 1). For both admissions and discharges, 65% (19/29) of virtual nursing encounters experienced at least one instance of fatigue by 15 minutes, followed by another spike at 30 and 45 minutes. When analyzing the preparation and miscellaneous tasks, we found similar 15-minute interval patterns.

In addition, 75% (22/29) of virtual nursing encounters experienced their first fatigue instance within 9.8 minutes during patient discharges and within 11.9 minutes during patient admissions, Figure 2. Half of virtual nursing encounters (15/29) showed a first fatigue instance within 5.7 minutes during patient discharges and within 3.2 minutes for patient admissions.

We also examined the relationship between patient acuity and fatigue. Of the 29 unique patient admission and discharge encounters, we found that 8 (28%) patients were considered low acuity, 15 (51%) were considered medium acuity, and 6 (21%) were considered high acuity.

Fatigue instances varied by patient acuity (Figure 3). High-acuity patients showed a steeper increase in fatigue than medium and low-acuity patients. Regardless of patient acuity, 75% (62/83) of encounters had their first instance of fatigue within 8.6 minutes.

## Discussion

### Principal Findings

For the first time, this study quantified the association between the virtual nursing workflow and virtual nurse cognitive fatigue. We report that screen time–related fatigue instances reach maximum levels over 15-minute intervals. This suggests the need to consider more frequent breaks between patient encounters to reduce cognitive fatigue for virtual nurses compared to the standard routine for bedside nurses. We also found that approximately 75% of virtual nurses experience their first fatigue instance after approximately 10 minutes, regardless of the encounter type or patient acuity. By comparison, our previous study suggested that cognitive fatigue occurs after 22 minutes of EHR use by physicians [17].

Suboptimal EHR interface designs and documentation burdens contribute to nurse fatigue [16,25-27]. Virtual nursing can reduce EHR time and the documentation burden for bedside nurses; however, it is paramount to avoid shifting these job stressors to virtual nurses. Virtual nurses who spend extended durations working in the EHR and documenting patient assessments may exhibit cognitive fatigue, a precursor to nurse burnout. This study provides actionable insights into the relationship between using virtual nursing and virtual nurses’ cognitive fatigue.

In general, planned and integrated work breaks have been shown to improve the well-being and performance of bedside nurses [28-30]. Our findings support a similar workflow design for virtual nurses that incorporates brief breaks every 15 minutes or between patient sessions. The type of break and its ability to achieve its goals is more important than the frequency of breaks. Prior studies show that breaks should be designed to enable virtual nurses to detach psychologically including active rest and the use of mental health applications [31-33].

For bedside nurses, having an opportunity to rest and physically remove oneself from the work environment can facilitate psychological detachment [31]. However, different work breaks may be needed for virtual nurses as their workflow is primarily computer-based and can become repetitive. One study found that an 8-minute-long virtual reality session during work breaks reduced fatigue among nurses working in high-intensity and cognitively engaging environments [34].

Future research should examine practical work break activities and their impact on virtual nurses’ fatigue, including whether different types of breaks (active vs passive) are more effective in reducing instances of fatigue. In addition, future research should use eye-tracking technology to assess the relationship between virtual nursing use and cognitive fatigue among bedside nurses. Although virtual nursing is an emerging care delivery model, there is a critical gap in knowledge around the effect of virtual nursing on organizational and nurse-level outcomes, including nurse burnout.

### Limitations

This study was limited by a small sample size of four virtual nurses at one hospital. The study recordings were affected by fluctuations in the virtual nurses’ shifts and virtual nurse availability, leading to some nurses having more recorded duration than others. This also resulted in recordings primarily being captured in the middle of virtual nursing work shifts. Future research should examine if instances of fatigue are varied during different times of the shift, including in the beginning and the end of the work shift. The study was limited to a single hospital using the same proficient electronic health record system. Future research should expand on both the nurse sample size as well as the number of institutions to better account for generalizability in the virtual nursing workflow. Additionally, as this study recorded encounters during the live clinical environment and for extended periods of time, eye-tracking accuracy was affected. To ensure an accurate dataset, our analysis only included eye-tracking instances where the percentage of valid pupil data was ≥10%.

To address some of the aforementioned limitations, future research should incorporate larger sample sizes, comparative analyses, and fatigue mitigation strategies to optimize virtual nurse work environments. Future work should incorporate the use of additional measures of cognitive fatigue to ensure accuracy, such as an additional eye-tracker or subjective fatigue surveys for validation. While the study examined patient acuity as a potential confounder, additional contributors to fatigue and confounders may have been unintentionally excluded. Future research should ensure that all contributing factors are addressed in both the recordings and subsequent analyses. In addition, future research should use longitudinal studies to assess the relationship between virtual nursing and virtual nurse fatigue over extended periods to determine potential long-term effects, including exhaustion and burnout. Finally, while the study team used a multifactor approach to assign patient acuity ratings given available data, future research avenues should examine how fatigue is affected by standardized patient acuity scales, such as the Emergency Severity Index.

### Conclusions

This cross-sectional study used eye-tracking methods to understand the effect of continuous screen time on virtual nurses’ cognitive fatigue during virtual nursing sessions. The findings emphasize that continuous screen time in virtual nursing tasks contributes to nurses’ cognitive fatigue and that specific workflow designs are required to reduce screen time–related fatigue. Health systems implementing virtual nursing should consider frequent breaks away from computer workstations to alleviate the effect of continuous screen time on virtual nurses’ well-being. These breaks could incorporate instances of active rest or the use of mental health applications, which have been shown to reduce burnout and fatigue in bedside nurses and other healthcare providers. Findings from this study have direct policy implications and suggestions for more effective workflow redesign.

## Abbreviations

EHR: electronic health record
IRB: Institutional Review Board
TOI: time(s) of interest
